# The Freedom of Clinicians and the Art of the Impossible

**DOI:** 10.5935/abc.20170163

**Published:** 2017-11

**Authors:** Hugo Madeira

**Affiliations:** Past-President of the Federation of the Portuguese Language Societies of Cardiology; Centro Cardiovascular da Universidade de Lisboa, Lisboa - Portugal

**Keywords:** Hypertension, Risk Factors, Blood Pressure / control & prevention, Primary Health Care

Systemic arterial hypertension - or simply hypertension, in casual language - is considered the major and most common risk factor for death and disability of non-communicable diseases.^1,2^ Its prevalence in Europe ranges from 30% to 45%.^3^ In the United States, two thirds of the adults older than 60 years are hypertensive.^4^ In South Asia and Sub-Saharan Africa, hypertension has increased rapidly.^5^ Recently, the world prevalence of hypertension was estimated at 31%.^6^

The last three decades have witnessed the development of several effective and safe drugs to treat hypertensives. However, although a blood pressure reduction by only 10 mm Hg in those patients is known to reduce the risk of cardiovascular death and stroke by 25-40% throughout life,^7^ the threshold value or target value to be achieved in hypertensive adults in general, and in the elderly in particular, is controversial. In addition, several patients remain poorly controlled despite treatment, without reaching the target values of the ESC/ESH Recommendations^3^ or those suggested as a result of the SPRINT study.^8^

Several guidelines/recommendations for the diagnosis and treatment of hypertension have been published by scientific societies or other international and national public agencies, without reaching absolute consensus. Regarding the systolic blood pressure levels originally proposed by the *5th Joint National Committee* (< 140 mmHg)^9^ and those emerging from the SPRINT study (< 120 mmHg), there is an indecision/decision range, and although it is believed that "lower blood pressure is better" for patients in general, the clinicians should decide.

Recommendations in medicine, originally clinical practice guides suggesting an approach for the management of difficult clinical situations, let clinicians free to adjust therapy according to the patient's specificity. For example, in case of hypertension, clinicians could decide upon a more "aggressive" therapy for younger patients, even if asymptomatic, or upon a more conservative one (admitting higher systolic blood pressure levels) for the elderly, supposedly - what is still a matter of discussion - more susceptible to complications from treatment itself.

That initial therapeutic flexibility has diminished, although not explicitly. The recommendations, written and edited based on studies not rarely different from the real world, began to define what clinicians should do in each circumstance, under penalty of their performance being considered poor clinical practice. Briefly, the "recommendations" became "guidelines", and the semantic change in Portuguese says a lot.

It is worth noting that attending physicians should always, taking into account their patients' characteristics - cardiovascular risk, general well-being, weaknesses and options - and weighing the drawbacks from occasional adverse effects of treatment, make the best decisions.

In this scenario, the guidelines now published were created, intended for the Federation of the Portuguese Language Societies of Cardiology (*Federação das Sociedades de Cardiologia de Língua Portuguesa* - FSCLP - www.fsclp.org). The FSCLP was created in 2014 aimed mainly at *"promoting the development of* *Cardiology to serve the population of countries and territories whose official language is Portuguese"* - (statutes, 4^th^ article). Prior to its foundation, Lusophone Cardiology Meetings were held in Cape Verde (2009) and Mozambique (2011). The first FSCLP Congress was held in Portugal in 2016, and the second one will be held in Brazil in November 2017.

In the already-mentioned statutes, the pathways to substantiate the major objective are succinctly enunciated, the most important being: to stimulate the study and investigation of the scientific issues related to cardiovascular disease; to analyze the social aspects of heart diseases and their prevention, as well as patient care; and to narrow the relationship between the physicians of Portuguese-speaking societies and communities dedicated to cardiology. Concisely, to develop Lusophone Cardiology.

To create more guidelines^10^ for the FSCLP that would not repeat what is already written seemed like an impossible challenge. Nevertheless, these Guidelines for *"Arterial Hypertension Management in Primary Heath Care in Portuguese Language Countries"* emerge valuable. Firstly, they depict accurately the reality of the Lusophone space, with its similarities and differences. Secondly, avoiding excessive observations, they do not leave essential aspects out. Thirdly - and decisively - they emphasize the importance of hypertension prevention and treatment in primary health care, which is, after all, their objective. Finally, they take into account the medical, social and economic characteristics of the space they are destined for.

In addition, these Guidelines^10^ published here have another very significant merit: they are the first scientific and pedagogical work by the FSCLP, which is something to be proud of. These Guidelines are aimed at meeting the goals of the FSCLP and at taking a big step towards the beginning of a *"continuous process, involving mainly educational actions, lifestyle changes and guaranteed access to medicines"* for hypertension, as stated in the document itself.

The authors of these Guidelines have outlined with Art what seemed Impossible.
